# Noncancer-Related Mortality in Randomized Clinical Trials: A Meta-Analysis

**DOI:** 10.1001/jamanetworkopen.2025.26990

**Published:** 2025-08-25

**Authors:** Jiayao Lei, Yunyang Deng, Mark Clements, Stephen Duffy, Peter Sasieni

**Affiliations:** 1 Department of Medical Epidemiology and Biostatistics, Karolinska Institutet, Stockholm, Sweden; 2 Department of Clinical Science, Intervention and Technology, Karolinska Institutet, Stockholm, Sweden; 3 Centre for Cancer Screening, Prevention, and Early Detection, Wolfson Institute of Population Health, Queen Mary University of London, London, United Kingdom

## Abstract

**Importance:**

Cancer screening is a critical tool in cancer control, reducing cancer-specific mortality. However, it also has potential harms, including overdiagnosis and overtreatment. Measuring the effect of screening based on all-cause mortality is insensitive to both benefits and harms and requires substantially large sample sizes. Understanding the impact of screening on noncancer-related (off-target) mortality is essential for evaluating its overall benefit.

**Objective:**

To assess the association between cancer screening and off-target mortality by comparing mortality rates between screened and unscreened populations based on randomized clinical trials (RCTs).

**Data Sources:**

The analysis examined all RCTs included in a previously published (August 28, 2023) meta-analysis of cancer screening trials that included the end point of all-cause mortality in addition to targeted cancer mortality.

**Study Selection:**

All RCTs included in the previous meta-analysis were included. The latest search in that meta analysis was conducted on October 12, 2022, with no language or publication date restrictions.

**Data Extraction and Synthesis:**

The study followed relevant portions of the Preferred Reporting Items for Systematic Reviews and Meta-Analyses (PRISMA) reporting guideline. Two authors independently extracted data, and a third author verified those data. Off-target mortality was analyzed using rate ratios (RRs) and 95% CIs via a fixed-effects model. Heterogeneity was assessed using the *I*^2^ statistic.

**Main Outcome and Measures:**

The primary outcome was off-target mortality, defined as deaths with a cause that was not the targeted cancer.

**Results:**

A total of 17 RCTs (8 of colorectal, 3 prostate, 3 lung, 2 breast, and 1 multiple cancers) including 1 305 924 participants with 18 508 192 person-years of follow-up were included. Screening did not significantly increase off-target mortality (RR, 1.00; 95% CI, 1.00-1.01); the overall increase in off-target mortality was 0.2% (95% CI, −0.5% to 0.9%). There was no evidence of heterogeneity between trials (*I*^2^ = 0.00%; Cochran *Q* = 14.96, *df* = 18; *P* = .66). The trial-specific RRs ranged from 0.89 (95% CI, 0.69-1.15) to 1.09 (95% CI, 0.98-1.22), with all 95% CIs including 1. Targeted cancer deaths accounted for 2.6% to 33.1% of all deaths, depending on the cancer type.

**Conclusions and Relevance:**

These findings show that randomization to cancer screening was not associated with more than a very small increase in noncancer-related mortality, with the 95% CI excluding an increase of greater than 1%. The findings emphasize the importance of evaluating targeted and off-target mortality separately rather than relying solely on all-cause mortality.

## Key Points

**Question:**

Are there differences in noncancer-related mortality between the screening and control arms of randomized clinical trials?

**Findings:**

In this meta-analysis of 17 randomized clinical trials including 1 305 924 participants, allocation to cancer screening was not associated with an increase in noncancer-related mortality. The overall increase in off-target mortality was 0.2%, but the difference was not statistically significant.

**Meaning:**

These findings highlight the importance of assessing targeted and off-target mortality separately rather than relying solely on all-cause mortality.

## Teaser

This meta-analysis assesses the association between cancer screening and noncancer-related mortality between screened and unscreened participants in randomized clinical trials.

## Introduction

Screening, including mammography for breast, prostate-specific antigen testing for prostate, fecal occult blood testing or endoscopy for colorectal, computed tomography for lung, and cytology and human papillomavirus testing for cervical cancer, is a component of cancer control. Cancer screening has been shown to reduce cancer-specific mortality; however, screening also causes harms through overdiagnoses and associated overtreatment.^1,2^

A recent meta-analyses by Bretthauer et al^3^ claimed that cancer screening does not save lives, except possibly for colorectal cancer screening with sigmoidoscopy. Concerns regarding the use of all-cause mortality for evaluating cancer screening have been raised previously.^4,5,6^ Furthermore, if screening does not extend life, it must be associated with increased off-target mortality to balance the reduction in cancer-specific mortality. Here, we assess the difference in off-target mortality between the arms of randomized clinical trials (RCTs) of cancer screening.

## Methods

In this meta-analysis, we did not conduct a new search but have included and reviewed all RCTs included in the meta-analysis by Bretthauer et al.^3^ They searched Medline and the Cochrane Library for reports and meta-analyses of RCTs comparing screening vs no screening with cause-specific and all-cause mortality as end points; the latest search was performed on October 12, 2022. Bretthauer et al^3^ were looking for RCTs of types of cancer screening that are generally recognized to reduce cancer-specific mortality. To this end, they did not include trials of outdated screening tests (eg, chest radiography for lung cancer). For breast screening, they excluded trials that started screening at age younger than 50 years and those excluded from a previous controversial meta-analysis.^7,8^ We followed relevant portions of the Preferred Reporting Items for Systematic Reviews and Meta-Analyses (PRISMA) reporting guideline.

We did not assess potential biases in each trial. For each trial, we collected data from the original publication (rather than from a meta-analysis). Two authors (J.L. and Y.D.) independently assessed each trial and extracted data, and a third author (P.S.) reviewed the extracted data.

### Statistical Analysis

Our end point was off-target mortality (ie, death other than from the targeted cancer). We reviewed each trial and extracted (1) numbers of individuals randomized to each arm, (2) number of off-target deaths (either directly or calculated by subtraction), and (3) person-years at risk during follow-up (sometimes calculated from the reported number of events and mortality rates). All numbers were retrieved based on intention-to-treat comparisons of individuals assigned to either screening or no screening. For studies for which the number of events or person-time was not directly available, methods used are detailed in the eMethods in Supplement 1.

Rate ratios (RRs) with 95% CIs and a fixed-effects model were used in our meta-analysis. We measured heterogeneity using both the *I*^2^ statistic and Cochran *Q* test. Two-sided *P* < .05 indicated statistical significance. In a sensitivity analysis, the effect sizes were pooled using a random-effects model. The data analysis was performed using the command meta in Stata, version 18.0 (StataCorp LLC).

## Results

### Trial Characteristics

We included 17 RCTs encompassing 1 305 924 participants with 18 508 192 person-year of follow-up^9,10,11,12,13,14,15,16,17,18,19,20,21,22,23,24,25^ (Table; eTable in Supplement 1). Two of the trials^13,25^ were split into 2 (each) for the meta-analysis. Eight issues were identified in the presented data from Bretthauer et al^3^ (eMethods in Supplement 1). Details on how data were derived in each of the trials in our study, if not directly available, are presented in the eMethods in Supplement 1.

**Table.** Characteristics of Randomized Clinical Trials Included in the Main Analyses

Source	Screening modality	Screening			No screening				
		Total, No.	Off-target deaths, No.	Person-years, No.	Total, No.	Off-target deaths, No.	Person-year, No.	Total deaths, No.	Target cancer-related deaths, No. (% of total deaths)
**Colorectal cancer**									
Bretthauer et al,^9^ 2022^a^	Colonoscopy	28 220	2964	260 788	56 365	5922	520 515	6079	157 (2.6)
Atkin et al,^10^ 2017^b^	Sigmoidoscopy	57 098	12 926	902 106	112 936	25 413	1 780 782	26 409	996 (3.8)
Holme et al,^11^ 2018^b^^,^^c^	Sigmoidoscopy	20 552	3687	291 075	78 126	12 903	1 006 828	13 433	530 (3.9)
Senore et al,^12^ 2021^b^	Sigmoidoscopy	17 136	2940	296 730	17 136	2 998	295 013	3155	157 (5.0)
Mandel et al,^13^ 1999	Biennial FOBT	15 587	5065	240 163	15 394	5009	237 420	5186	177 (3.4)
Scholefield et al,^14^ 2012	Biennial FOBT	76 056	39 505	1 296 712	75 919	39 250	1 296 614	40 550	1300 (3.2)
Kronborg et al,^15^ 2004	Biennial FOBT	30 967	11 778	431 190	30 966	11 769	430 755	12 248	479 (3.9)
Lindholm et al,^16^ 2008	Biennial FOBT	34 144	10 339	471 072	34 164	10 132	471 980	10 432	300 (2.9)
Mandel et al,^13^ 1999	Annual FOBT	15 570	5115	240 325	15 394	5009	237 420	5186	177 (3.4)
Subtotal	NA	295 330	94 319	4 430 161	436 400	118 405	6 277 327	122 678	4273 (3.5)
**Prostate cancer**									
Martin et al,^17^ 2018^d^	PSA testing	25 324	3331	247 796	29 342	3698	280 187	3785	87 (2.3)
Schröder et al,^18^ 2014	PSA testing	72 891	15 014	825 018	89 352	18 563	1 011 192	19 108	545 (2.9)
Lundgren et al,^19^ 2018^e^	PSA testing	2400	1334	36 960	25 081	13 846	386 247	14 703	857 (5.8)
Subtotal	NA	100 615	19 679	1 109 774	143 775	36 107	1 677 626	37 596	1489 (4.0)
**Lung cancer**									
de Koning et al,^20^ 2020	Lung CT	6583	708	62 298	6612	650	62 484	860	210 (24.4)
Wille et al,^21^ 2016	Lung CT	2052	126	19 439	2052	125	19 547	163	38 (23.3)
Paci et al,^22^ 2017	Lung CT	1 613	111	14 658	1593	121	14 247	181	60 (33.1)
Subtotal	NA	10 248	945	96 395	10 257	896	96 278	1204	308 (25.6)
**Breast cancer**									
Miller et al,^23^ 2014^f^	Mammography	44 925	4289	983 858	44 910	4183	983 529	4688	505 (10.8)
Tabar et al,^24^ 1989^g^	Mammography	46 897	3874	369 441	33 074	2766	263 323	2878	112 (3.9)
Subtotal	NA	91 822	8163	1 353 299	77 984	6949	1 246 852	7566	617 (8.2)
**4-Cancer**									
Pinsky et al,^25^ 2019^h^	Lung, colorectal, and prostate cancer (among male participants)	38 340	11 777	601 370	38 338	11 926	594 077	13 842	1916 (13.8)
Pinsky et al,^25^ 2019^h^	Lung, ovarian, and colorectal cancer (among female participants)	39 103	7789	633 616	39 106	7625	628 837	8810	1185 (13.5)
Subtotal	NA	77 443	19 566	1 234 986	77 444	19 551	1 222 914	22 652	3101 (13.7)

Abbreviations: CT, computed tomography; FOBT, fecal occult blood test; NA, not applicable; PSA, prostate-specific antigen.

^a^ Person-time was calculated based on the number of individuals at risk, as shown in the Kaplan-Meier plot from Bretthauer et al.^3^

^b^ Numbers derived from the meta-analyses by Juul et al,^26^ and each of the trials are included separately for analyses.

^c^ The person-time for the control arm was calculated using the number of total deaths divided by the age-standardized all-cause mortality rate.

^d^ All values were adjusted for the variance inflation factor due to cluster randomization.

^e^ The event numbers for the control arm were derived from the source population in the original publication, which corresponded to the actual control arm values based on the reported rates.

^f^ Person-time was calculated as the number of individuals in each arm multiplied by the mean follow-up period of 21.9 years.

^g^ Person-time was calculated as the sum of the midpopulation at risk, stratified by county, study group, age, and time since randomization.

^h^ Data were extracted separately for male and female participants, depending on cancer type. For female participants, ovarian cancer–related death was not included in the off-target deaths. For both male and female participants, lung cancer deaths were treated as on-target even though the screening was by radiography.

### Off-Target Mortality

The overall increase in off-target mortality was 0.2% (95% CI, −0.5% to 0.9%). Trial-specific RRs ranged from 0.89 (95% CI, 0.69-1.15)^22^ to 1.09 (95% CI, 0.98-1.22),^20^ and the individual 95% CIs all included 1 (heterogeneity: *I*^2^ = 0.00%; Cochran *Q* = 14.96; *df* = 18; *P* = .66). Overall, off-target mortality for the screening population was not statistically different than that for the no-screening population (RR, 1.00; 95% CI, 1.00-1.01), corresponding to an increase in off-target mortality of 0.2% (95% CI, −0.5% to 0.9%) (Figure). The 95% CI indicates that, at most, off-target mortality was increased by 0.9%. By using a random-effects model, we found the estimate for the off-target mortality was identical to the main result (eFigure in Supplement 1).

**Figure.**
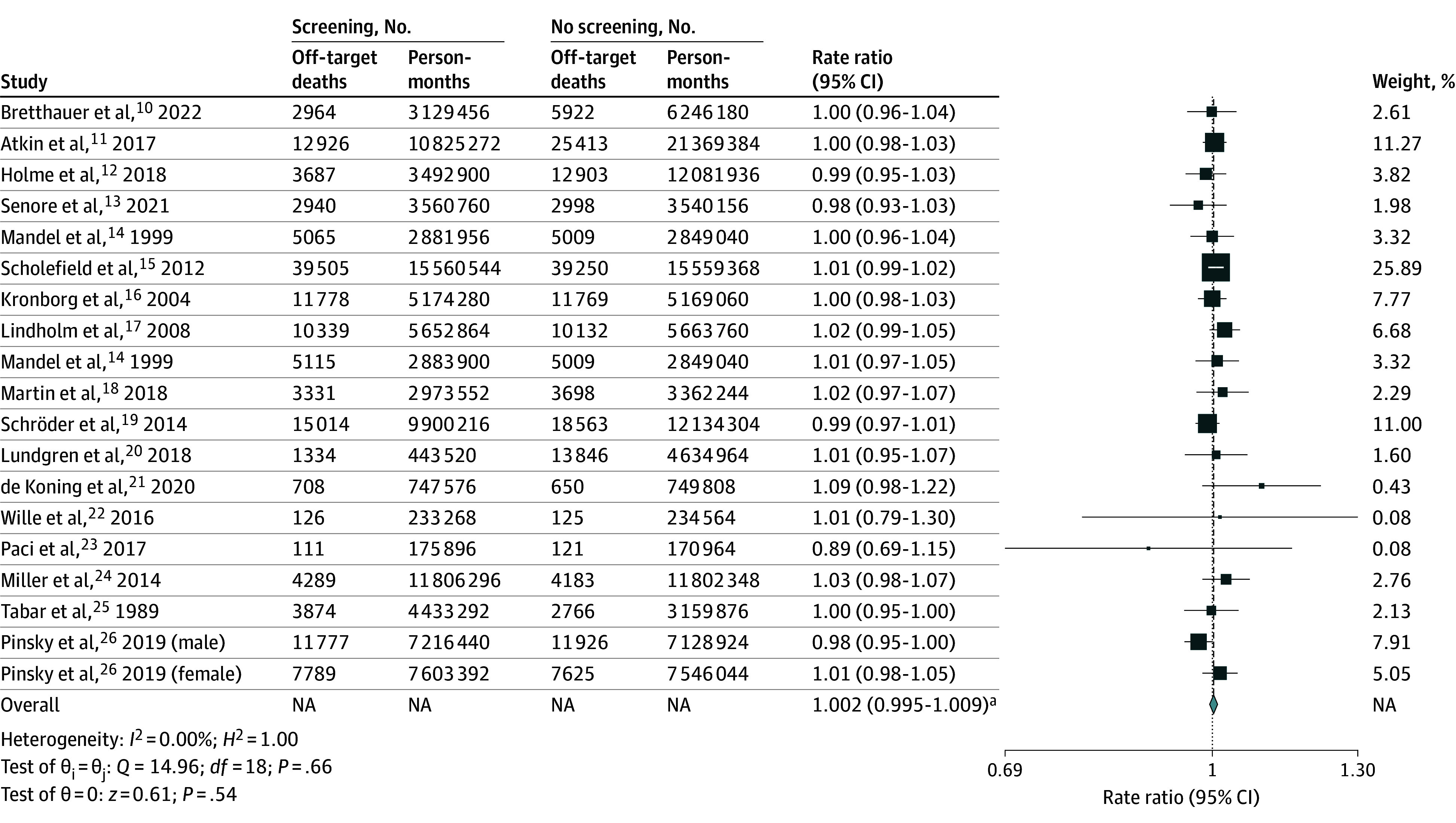
Relative Risk of Noncancer Mortality in Included Randomized Clinical Trials The size of the box represents the weight that each study contributes to the overall pooled estimate. NA indicates not applicable. ^a^Since the width of the confidence interval is less than 0.02, the result is provided to 3 decimal places.

Deaths from the targeted cancer represented 2.6% (157 of 6079 participants)^9^ to 5.0% (157 of 3155 participants)^12^ of all deaths in the control arm of bowel cancer screening trials, 2.3% (87 of 3785 participants)^17^ to 5.8% (857 of 14 703 participants)^19^ for prostate-specific antigen testing, 23.3% (38 of 163 participants)^21^ to 33.1% (60 of 181 participants)^22^ in targeted lung screening trials, 3.9% (112 of 2878 participants)^24^ to 10.8% (505 of 4688 participants)^23^ in mammography screening trials, and 13.5% (1185 of 8810 female participants) to 13.8% (1916 of 13 842 male participants) in the Prostate, Lung, Colorectal, Ovary trial^25^ (Table). Based on data from the Table, a 20% reduction in targeted mortality would correspond to an approximately 0.7% (0.2 × 3.5%), 0.8% (0.2 × 4.0%), 5.1% (0.2 × 25.6%), 1.6% (0.2 × 8.2%), and 2.7% (0.2 × 13.7%) reduction in all-cause mortality, respectively. For breast and targeted lung screening, the reduction in on-target deaths greatly exceeded the maximal expected increase in off-target deaths.

## Discussion

This meta-analysis found that on average, cancer screening increases off-target mortality by approximately 1% (upper 95% CI). Whereas Bretthauer et al^3^ emphasized the lack of empirical RCT evidence for cancer screening saving lives, we found that the same trials provide strong evidence of the lack of any nontrivial effect of screening on off-target mortality. Including other cancer screening RCTs^27^ would mostly narrow the 95% CI. Thus, where screening has been shown to have a beneficial effect on targeted cancer mortality, combining that result with the lack of a substantial association with off-target mortality means that one could infer with reasonable confidence that screening must be saving lives.

Combining the actual numbers of target-specific deaths with off-target deaths mixes 2 outcomes, which are strikingly heterogeneous, and conceals important information. Looking at all-cause mortality in a meta-analysis of screening trials is problematic because one would not expect any effect in any trial in which there was no effect on targeted mortality (but only including trials in which there was a significant effect on targeted mortality would bias the results). Our findings highlighted the importance of quantifying the benefits and harms of screening separately and using appropriate methods to combine these to estimate the overall impact on a composite end point such as all-cause mortality.

Screening could potentially cause off-target mortality through 2 distinct routes. Investigation of false-positive screening results or treatment of screen-detected cancers may expose patients to additional surgery, radiation, or chemotherapy, which carry risks of complications and could lead to premature death.^28^ For instance, the increased use of radiation to treat screen-detected breast cancer might lead to an increase in cardiovascular deaths. Indeed, a weakness of the approach taken here is that we combined off-target results from many different types of screening, whereas the potential off-target impact of different types of screening may be qualitatively different. To assess whether patients with screen-detected cancers simply had a different cause of death recorded (with no extension of life), Tabar et al^29^ studied all-cause mortality in those with breast cancer. The other potential route to harm is a change in behavior resulting from a negative screening test. The most likely example is for individuals to ignore symptoms and delay diagnosis following a normal screen, but any such effect would have an impact on cancer-specific mortality. It is possible that a negative screening result may encourage risk-taking (eg, an increase in smoking or unhealthy eating), but there are scant empirical data to support such a scenario.

The aim of this study was not to be a comprehensive systematic review of cancer screening. Rather, our aim was to consider more leveraged analyses for studying the ultimate harm of screening, ie, an increase in off-target mortality. We have shown that studying off-target and targeted mortality separately could be more informative than studying all-cause mortality directly. Indeed, having found that there was at most a very small increase in off-target mortality associated with screening, it is clear that one needs to examine targeted mortality to conclude that screening saves lives.

### Limitations

Limitations of the study include reliance on preselected trials from an existing publication rather than conducting a comprehensive literature review. The trials included were heterogeneous in many factors, including compliance with screening, contamination of the control arm, length of follow-up, and screening modalities. Besides, the accuracy of the cause-of-death measurement may vary depending on the method used for ascertainment in individual trials, but note that misattribution that leads to an overestimate of the benefit in terms of cancer-specific mortality may also lead to overestimation of the harm in terms of off-target mortality and vice versa. Additionally, several of the included trials were conducted decades ago, using cancer screening technologies that differ from those in current practice. For instance, most breast cancer screening trials used film or early digital mammography, whereas certain programs use digital breast tomosynthesis, which has demonstrated improved cancer detection rates and reduced recall rates.^30^ We need to be mindful about the changes in screening modalities over time when interpreting our findings for current practice. Changes in cancer treatment over time are also important to consider. Advances in therapy, particularly those that differentially affect early- and late-stage cancers, may influence the benefits of screening. Improvements in treatment safety, such as more targeted radiotherapy, may reduce the harms associated with overdiagnosis.

## Conclusions

In this meta-analysis of cancer screening and off-target mortality, our findings support, at most, a minimal increase in off-target mortality caused by cancer screening. Understanding the potential harms related to overdiagnosis and overtreatment remains crucial, but the overall balance of risk appears to favor the continuation of cancer screening practices because screening saves lives.

## Notes

Supplement 1. **eTable.** List of Randomized Clinical Trials Included in the Meta-Analysis **eMethods.** Items With Issues in the Data by Bretthauer et al **eFigure.** Random Effects on Relative Risk of Noncancer Mortality in Included Randomized Clinical Trials

Supplement 2.
Data Sharing Statement

## References

[1] Zielonke N, Gini A, Jansen EEL, ; EU-TOPIA consortium. Evidence for reducing cancer-specific mortality due to screening for breast cancer in Europe: a systematic review. Eur J Cancer. 2020;127:191-206. doi:10.1016/j.ejca.2019.12.010 31932175

[2] Srivastava S, Koay EJ, Borowsky AD, . Cancer overdiagnosis: a biological challenge and clinical dilemma. Nat Rev Cancer. 2019;19(6):349-358. doi:10.1038/s41568-019-0142-8 31024081 PMC8819710

[3] Bretthauer M, Wieszczy P, Løberg M, . Estimated lifetime gained with cancer screening tests: a meta-analysis of randomized clinical trials. JAMA Intern Med. 2023;183(11):1196-1203. doi:10.1001/jamainternmed.2023.3798 37639247 PMC10463170

[4] DeGregori J, Pharoah P, Sasieni P, Swanton C. Cancer screening, surrogates of survival, and the soma. Cancer Cell. 2020;38(4):433-437. doi:10.1016/j.ccell.2020.09.003 32946774

[5] Dobbin KK, Ebell M. Should we expect all-cause mortality reductions in large screening studies? Br J Gen Pract. 2018;68(671):290-291. doi:10.3399/bjgp18X696545 29853594 PMC6002009

[6] Sasieni PD, Wald NJ. Should a reduction in all-cause mortality be the goal when assessing preventive medical therapies? Circulation. 2017;135(21):1985-1987. doi:10.1161/CIRCULATIONAHA.116.023359 28533316

[7] Gøtsche PC, Olsen O. Is screening for breast cancer with mammography justifiable? Lancet. 2000;355(9198):129-134. doi:10.1016/S0140-6736(99)06065-1 10675181

[8] Duffy SW, Tabar L, Chen THH, Yen AMF, Dean PB, Smith RA. A plea for more careful scholarship in reviewing evidence: the case of mammographic screening. BJR Open. 2023;5(1):20230041. doi:10.1259/bjro.20230041 37942497 PMC10630970

[9] Bretthauer M, Løberg M, Wieszczy P, ; NordICC Study Group. Effect of colonoscopy screening on risks of colorectal cancer and related death. N Engl J Med. 2022;387(17):1547-1556. doi:10.1056/NEJMoa2208375 36214590

[10] Atkin W, Wooldrage K, Parkin DM, . Long term effects of once-only flexible sigmoidoscopy screening after 17 years of follow-up: the UK Flexible Sigmoidoscopy Screening randomised controlled trial. Lancet. 2017;389(10076):1299-1311. doi:10.1016/S0140-6736(17)30396-3 28236467 PMC6168937

[11] Holme Ø, Løberg M, Kalager M, . Long-term effectiveness of sigmoidoscopy screening on colorectal cancer incidence and mortality in women and men: a randomized trial. Ann Intern Med. 2018;168(11):775-782. doi:10.7326/M17-1441 29710125 PMC6853067

[12] Senore C, Riggi E, Armaroli P, ; SCORE Working Group. Long-term follow-up of the Italian Flexible Sigmoidoscopy Screening Trial. Ann Intern Med. 2022;175(1):36-45. doi:10.7326/M21-0977 34748376

[13] Mandel JS, Church TR, Ederer F, Bond JH. Colorectal cancer mortality: effectiveness of biennial screening for fecal occult blood. J Natl Cancer Inst. 1999;91(5):434-437. doi:10.1093/jnci/91.5.434 10070942

[14] Scholefield JH, Moss SM, Mangham CM, Whynes DK, Hardcastle JD. Nottingham trial of faecal occult blood testing for colorectal cancer: a 20-year follow-up. Gut. 2012;61(7):1036-1040. doi:10.1136/gutjnl-2011-300774 22052062

[15] Kronborg O, Jørgensen OD, Fenger C, Rasmussen M. Randomized study of biennial screening with a faecal occult blood test: results after nine screening rounds. Scand J Gastroenterol. 2004;39(9):846-851. doi:10.1080/00365520410003182 15513382

[16] Lindholm E, Brevinge H, Haglind E. Survival benefit in a randomized clinical trial of faecal occult blood screening for colorectal cancer. Br J Surg. 2008;95(8):1029-1036. doi:10.1002/bjs.6136 18563785

[17] Martin RM, Donovan JL, Turner EL, ; CAP Trial Group. Effect of a low-intensity PSA-based screening intervention on prostate cancer mortality: the CAP randomized clinical trial. JAMA. 2018;319(9):883-895. doi:10.1001/jama.2018.0154 29509864 PMC5885905

[18] Schröder FH, Hugosson J, Roobol MJ, ; ERSPC Investigators. Screening and prostate cancer mortality: results of the European Randomised Study of Screening for Prostate Cancer (ERSPC) at 13 years of follow-up. Lancet. 2014;384(9959):2027-2035. doi:10.1016/S0140-6736(14)60525-0 25108889 PMC4427906

[19] Lundgren PO, Kjellman A, Norming U, Gustafsson O. Long-term outcome of a single intervention population based prostate cancer screening study. J Urol. 2018;200(1):82-88.. doi:10.1016/j.juro.2018.01.080 29408619

[20] de Koning HJ, van der Aalst CM, de Jong PA, . Reduced lung-cancer mortality with volume CT screening in a randomized trial. N Engl J Med. 2020;382(6):503-513. doi:10.1056/NEJMoa1911793 31995683

[21] Wille MM, Dirksen A, Ashraf H, . Results of the randomized Danish Lung Cancer Screening Trial with focus on high-risk profiling. Am J Respir Crit Care Med. 2016;193(5):542-551. doi:10.1164/rccm.201505-1040OC 26485620

[22] Paci E, Puliti D, Lopes Pegna A, ; the ITALUNG Working Group. Mortality, survival and incidence rates in the ITALUNG randomised lung cancer screening trial. Thorax. 2017;72(9):825-831. doi:10.1136/thoraxjnl-2016-209825 28377492

[23] Miller AB, Wall C, Baines CJ, Sun P, To T, Narod SA. Twenty five year follow-up for breast cancer incidence and mortality of the Canadian National Breast Screening Study: randomised screening trial. BMJ. 2014;348:g366. doi:10.1136/bmj.g366 24519768 PMC3921437

[24] Tabar L, Fagerberg G, Duffy SW, Day NE. The Swedish two county trial of mammographic screening for breast cancer: recent results and calculation of benefit. J Epidemiol Community Health. 1989;43(2):107-114. doi:10.1136/jech.43.2.107 2512366 PMC1052811

[25] Pinsky PF, Miller EA, Zhu CS, Prorok PC. Overall mortality in men and women in the randomized Prostate, Lung, Colorectal, and Ovarian Cancer Screening Trial. J Med Screen. 2019;26(3):127-134. doi:10.1177/0969141319839097 30943843

[26] Juul FE, Cross AJ, Schoen RE, . 15-Year benefits of sigmoidoscopy screening on colorectal cancer incidence and mortality: a pooled analysis of randomized trials. Ann Intern Med. 2022;175(11):1525-1533. doi:10.7326/M22-0835 36215714

[27] Feng X, Zahed H, Onwuka J, . Cancer stage compared with mortality as end points in randomized clinical trials of cancer screening: a systematic review and meta-analysis. JAMA. 2024;331(22):1910-1917. doi:10.1001/jama.2024.5814 38583868 PMC11000135

[28] Lu D, Andersson TML, Fall K, . Clinical diagnosis of mental disorders immediately before and after cancer diagnosis: a nationwide matched cohort study in Sweden. JAMA Oncol. 2016;2(9):1188-1196. doi:10.1001/jamaoncol.2016.0483 27124325

[29] Tabar L, Duffy SW, Yen MF, . All-cause mortality among breast cancer patients in a screening trial: support for breast cancer mortality as an end point. J Med Screen. 2002;9(4):159-162. doi:10.1136/jms.9.4.159 12518005

[30] Marinovich MI, Hunter KE, Macaskill P, Houssami N. Breast cancer screening tomosynthesis or mammography: a meta-analysis of cancer detection and recall. J Natl Cancer Inst. 2018;110(9):942-949. doi:10.1093/jnci/djy121 30107542

