# Supplementary material for: Noncancer-Related Mortality in Randomized Clinical Trials: A Meta-Analysis
Source: JAMA Netw Open. 2025 Aug 25;8(8):e2526990. doi: 10.1001/jamanetworkopen.2025.26990 (PMC12379107; doi:10.1001/jamanetworkopen.2025.26990)
Supplement: Supplement 1. — eTable. List of Randomized Clinical Trials Included in the Meta-Analysis eMethods. Items With Issues in the Data by Bretthauer et al eFigure. Random Effects on Relative Risk of Noncancer Mortality in Included Randomized Clinical Trials [file jamanetwopen-e2526990-s001.pdf]

Supplemental Online Content

Lei J, Deng Y, Clements M, Duffy S, Sasieni P. Noncancer-related mortality in randomized clinical trials: a meta-analysis. *JAMA Netw Open*. 2025;8(8):e2526990. doi:10.1001/jamanetworkopen.2025.26990

eTable. List of Randomized Clinical Trials Included in the Meta-Analysis

eMethods. Items With Issues in the Data by Bretthauer et al

eFigure. Random Effects on Relative Risk of Noncancer Mortality in Included Randomized Clinical Trials

This supplemental material has been provided by the authors to give readers additional information about their work.

© 2025 Lei J et al. *JAMA Network Open*.

eTable. List of Randomized Clinical Trials Included in the Meta-Analysis

ID	Publications
1	Bretthauer M, Løberg M, Wieszczy P, et al. Effect of Colonoscopy Screening on Risks of Colorectal Cancer and Related Death. N Engl J Med. 2022;387(17):1547-1556. doi: 10.1056/NEJMoa2208375.
2	Atkin W, Wooldrage K, Parkin DM, et al. Long term effects of once-only flexible sigmoidoscopy screening after 17 years of follow-up: the UK Flexible Sigmoidoscopy Screening randomised controlled trial. Lancet. 2017;389(10076):1299-1311. doi: 10.1016/S0140-6736(17)30396-3.
3	Holme Ø, Løberg M, Kalager M, et al. Long-Term Effectiveness of Sigmoidoscopy Screening on Colorectal Cancer Incidence and Mortality in Women and Men: A Randomized Trial. Ann Intern Med. 2018;168(11):775-782. doi: 10.7326/M17-1441.
4	Senore C, Riggi E, Armaroli P, et al. Long-Term Follow-up of the Italian Flexible Sigmoidoscopy Screening Trial. Ann Intern Med. 2022;175(1):36-45. doi: 10.7326/M21-0977.
5	Mandel JS, Church TR, Ederer F, Bond JH. Colorectal cancer mortality: effectiveness of biennial screening for fecal occult blood. J Natl Cancer Inst. 1999;91(5):434-7. doi: 10.1093/jnci/91.5.434.
6	Scholefield JH, Moss SM, Mangham CM, Whynes DK, Hardcastle JD. Nottingham trial of faecal occult blood testing for colorectal cancer: a 20-year follow-up. Gut. 2012;61(7):1036-40. doi: 10.1136/gutjnl-2011-300774.
7	Kronborg O, Jørgensen OD, Fenger C, Rasmussen M. Randomized study of biennial screening with a faecal occult blood test: results after nine screening rounds. Scand J Gastroenterol. 2004 Sep;39(9):846-51. doi: 10.1080/00365520410003182.
8	Lindholm E, Brevinge H, Haglind E. Survival benefit in a randomized clinical trial of faecal occult blood screening for colorectal cancer. Br J Surg. 2008;95(8):1029-36. doi: 10.1002/bjs.6136.
9	Martin RM, Donovan JL, Turner EL, et al. Effect of a Low-Intensity PSA-Based Screening Intervention on Prostate Cancer Mortality: The CAP Randomized Clinical Trial. JAMA. 2018;319(9):883-895. doi: 10.1001/jama.2018.0154.
10	Schröder FH, Hugosson J, Roobol MJ, et al. Screening and prostate cancer mortality: results of the European Randomised Study of Screening for Prostate Cancer (ERSPC) at 13 years of follow-up. Lancet. 2014;384(9959):2027-35. doi: 10.1016/S0140-6736(14)60525-0.

© 2025 Lei J et al. *JAMA Network Open*.

ID	Publications
11	Lundgren PO, Kjellman A, Norming U, Gustafsson O. Long-Term Outcome of a Single Intervention Population Based Prostate Cancer Screening Study. J Urol. 2018;200(1):82-88. doi: 10.1016/j.juro.2018.01.080.
12	de Koning HJ, van der Aalst CM, de Jong PA, et al. Reduced Lung-Cancer Mortality with Volume CT Screening in a Randomized Trial. N Engl J Med. 2020;382(6):503-513. doi: 10.1056/NEJMoa1911793.
13	Wille MM, Dirksen A, Ashraf H, et al. Results of the Randomized Danish Lung Cancer Screening Trial with Focus on High-Risk Profiling. Am J Respir Crit Care Med. 2016;193(5):542-51. doi: 10.1164/rccm.201505-1040OC.
14	Paci E, Puliti D, Lopes Pegna A, et al. Mortality, survival and incidence rates in the ITALUNG randomised lung cancer screening trial. Thorax. 2017;72(9):825-831. doi: 10.1136/thoraxjnl-2016-209825.
15	Miller AB, Wall C, Baines CJ, Sun P, To T, Narod SA. Twenty five year follow-up for breast cancer incidence and mortality of the Canadian National Breast Screening Study: randomised screening trial. BMJ. 2014 Feb 11;348:g366. doi: 10.1136/bmj.g366.
16	Tabar L, Fagerberg G, Duffy SW, Day NE. The Swedish two county trial of mammographic screening for breast cancer: recent results and calculation of benefit. J Epidemiol Community Health. 1989;43(2):107-14. doi: 10.1136/jech.43.2.107.
17	Pinsky PF, Miller EA, Zhu CS, Prorok PC. Overall mortality in men and women in the randomized Prostate, Lung, Colorectal, and Ovarian Cancer Screening Trial. J Med Screen. 2019 Sep;26(3):127-134. doi: 10.1177/0969141319839097.

© 2025 Lei J et al. *JAMA Network Open*.

eMethods. Items With Issues in the Data by Bretthauer et al

Items with issues presented in Table 1 from Bretthauer M, Wieszczy P, Løberg M, et al. Estimated lifetime gained with cancer screening tests: a meta-analysis of randomized clinical trials. *JAMA Intern Med*. 2023;183(11):1196-1203. doi:10.1001/jamainternmed.2023.3798

Eight of the 22 rows of Table 1 from Bretthauer et al have issues that we have identified.

1. The paper by Juul et al is a pooled analyses of four randomized controlled trials. However, the individual trials are presented separately in the Table. Even though some of the data from the individual trials are excluded from Juul, if one were to use all the data from Table 1 in a meta-analysis one would be double counting some of the participants.
2. The Table cites Segnan et al, but the data are from a later publication on the same trial by Senore et al which includes longer follow-up.
3. Rates in Holme et al. Table 1 of Bretthauer et al presents the numbers of events and the numbers of individuals in each arm (as well as the mean duration of follow-up). The suggestion is that these can be used to calculate the rates in each arm. However, because (by design) the randomization ratios are different in different ages groups this would give the wrong answer. Since the proportion of younger participants randomised to screening is smaller than in older participants, the expected (crude) mortality rates (in the absence of any intervention) would be greater in those randomised to intervention than in those randomised to control. If one were to calculate the all-cause mortality rate ratio based on the numbers in the Table, the result would be wrong.
4. The Table refers to the 2002 paper by Jorgensen et al 13 years after the trial started, but cites data from the 2009 paper by Kronborg et al reporting after 17 years of the same trial.
5. Numbers of events in the control arm of Lundgren et al. Here the issue stems from the Lundgren paper. A column is headed "source population": it correctly includes the total number of participants in the study, but it records the numbers of events (prostate cancer, deaths, and prostate cancer deaths) from the control arm only. That this is the case can be worked out by calculating the rate ratios presented in the next column (labelled "all invited"). Using these data in meta-analysis, others (Juul et al and Bretthauer et al) subtracted the number of events in those invited from the number in the column labelled "source population" and thereby use a number of events in the control arm that is substantially too small.
6. The Table reports deaths from the part of the Canadian breast cancer screening trial in women aged 50-59. But the paper they cite, Miller et al 2014, is for women aged 40-59 and provides no breakdown by age at randomisation. Miller et al did publish just on women aged 50-59 (JNCI 2000) 14 years earlier, but even at that time there were more breast cancer deaths than reported in this meta-analysis.
7. In citing data from Tabar et al, they use data on deaths "from causes other than breast cancer" (Table 7) and cite them as deaths from any causes. Additionally, the number of deaths from all causes in the screening arm of Ostergotland (in women aged 50-69 at

© 2025 Lei J et al. *JAMA Network Open*.

randomisation) is cited as 1728 but the number of non-breast cancer deaths in Tabar et al is 1919. The difference (191) is the number of deaths in both years 6-7 and in years 7-8. Perhaps Bretthauer et al only counted 191 once.

8. Table 1 cites Andriole et al 2012 (13 years of follow-up) for the prostate component of the PLCO trial, but uses data from Pinsky et al 2017 with 15 years of follow-up.

© 2025 Lei J et al. *JAMA Network Open*.

eFigure. Random Effects on Relative Risk of Noncancer Mortality in Included Randomized Clinical Trials

Study	Screening Off-target deaths	Screening person-month	No screening Off-target deaths	No screening person-month	Rate Ratio with 95% CI	Weight (%)
Bretthauer et al, 2022	2,964	3,129,456	5,922	6,246,180	0.999 [0.956, 1.044]	2.60
Atkin et al, 2017	12,926	10,825,272	25,413	21,369,384	1.004 [0.983, 1.026]	11.29
Holme et al, 2018	3,687	3,492,900	12,903	12,081,936	0.988 [0.953, 1.025]	3.78
Senore et al, 2021	2,940	3,560,760	2,998	3,540,156	0.975 [0.927, 1.026]	1.96
Mandel et al, 1999	5,065	2,881,956	5,009	2,849,040	1.000 [0.961, 1.039]	3.32
Scholefield et al, 2012	39,505	15,560,544	39,250	15,559,368	1.006 [0.992, 1.021]	25.93
Kronborg et al, 2004	11,778	5,174,280	11,769	5,169,060	1.000 [0.975, 1.026]	7.77
Lindholm et al, 2008	10,339	5,652,864	10,132	5,663,760	1.022 [0.995, 1.051]	6.75
Mandel et al, 1999	5,115	2,883,900	5,009	2,849,040	1.009 [0.970, 1.049]	3.34
Martin et al, 2018	3,331	2,973,552	3,698	3,362,244	1.018 [0.972, 1.067]	2.31
Schroder et al, 2014	15,014	9,900,216	18,563	12,134,304	0.991 [0.970, 1.013]	10.94
Lundgren et al, 2018	1,334	443,520	13,846	4,634,964	1.007 [0.952, 1.065]	1.61
de Koning et al, 2020	708	747,576	650	749,808	1.092 [0.982, 1.215]	0.45
Wille et al, 2016	126	233,268	125	234,564	1.014 [0.791, 1.298]	0.08
Paci et al, 2017	111	175,896	121	170,964	0.892 [0.689, 1.154]	0.08
Miller et al, 2014	4,289	11,806,296	4,183	11,802,348	1.025 [0.982, 1.070]	2.79
Tabar et al, 1989	3,874	4,433,292	2,766	3,159,876	0.998 [0.951, 1.048]	2.13
Pinsky et al, 2019 (male)	11,777	7,216,440	11,926	7,128,924	0.976 [0.951, 1.001]	7.81
Pinsky et al, 2019 (female)	7,789	7,603,392	7,625	7,546,044	1.014 [0.982, 1.046]	5.08
**Overall**					1.002 [0.995, 1.009]	

Heterogeneity: $\tau^2 = 0.00$, $I^2 = 0.05\%$, $H^2 = 1.00$

Test of $\theta_i = \theta_j$: $Q(18) = 14.96$, $p = 0.66$

Test of $\theta = 0$: $z = 0.61$, $p = 0.54$

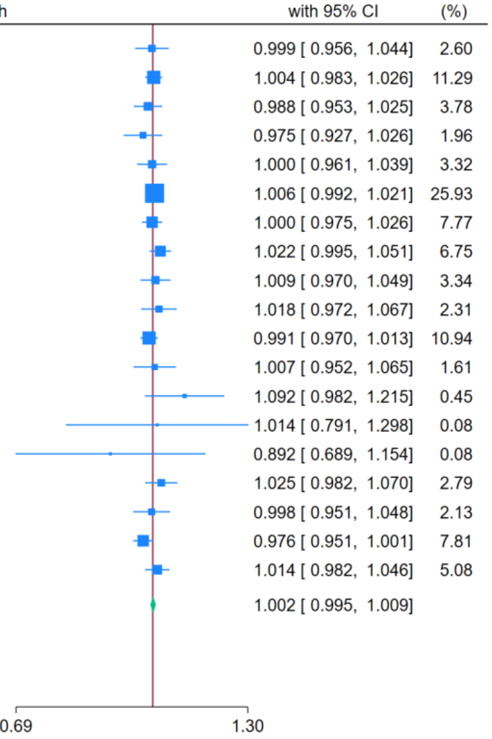

Random-effects REML model

© 2025 Lei J et al. *JAMA Network Open*.
