# Supplementary material for: Noncancer-Related Mortality in Randomized Clinical Trials: A Meta-Analysis
Source: JAMA Netw Open. 2025 Aug 25;8(8):e2526990. doi: 10.1001/jamanetworkopen.2025.26990 (PMC12379107; doi:10.1001/jamanetworkopen.2025.26990)
Supplement: Supplement 2. — Data Sharing Statement [file jamanetwopen-e2526990-s002.pdf]

Data Sharing Statement

Lei. Noncancer-Related Mortality in Randomized Clinical Trials. *JAMA Netw Open*. Published August 14, 2025. doi:10.1001/jamanetworkopen.2025.26990

Data
Data available: Yes
Data types: Other (please specify)
Additional Information: Summary data extracted from each trial
How to access data: As Supplementary material with the publication
When available: With publication

Supporting Documents
Document types: Statistical/analytic code
How to access documents: As Supplementary material with the publication
When available: With publication

Additional Information
Who can access the data: Anyone
Types of analyses: Any
Mechanisms of data availability: Public domain
Any additional restrictions: None
